# A commentary on ‘Performance of CT-based deep learning in diagnostic assessment of suspicious lateral lymph nodes in papillary thyroid cancer: a prospective diagnostic study’

**DOI:** 10.1097/JS9.0000000000000864

**Published:** 2023-11-07

**Authors:** Jiangming Qu, Ruichen Gao, Yu Chen

**Affiliations:** a Department of Radiology, Peking Union Medical College Hospital; b Peking Union Medical College, Chinese Academy of Medical Sciences, Beijing, People’s Republic of China

*Dear Editor*,

We read the article recently published in an upcoming issue of *International Journal of Surgery* by Zheng *et al*.^1^ with great interest. The authors conducted a prospective diagnostic study involving external validation to assess the efficacy of a 3D ResNet-based ensemble model, which utilizes contrast-enhanced computed tomography (CT) to differentiate between benign and metastatic lateral lymph nodes (LNs) from the suspicious LNs in papillary thyroid cancer (PTC). This innovative deep learning model is both noninvasive and time-efficient, presenting a viable approach for surgical planning before lateral neck dissection of PTC. However, we would like to discuss several concerns regarding the study.

First, unlike prior research employing deep learning models to analyze all detected LNs, this study specifically focused on LNs already stratified using CT-based criteria similar to a risk stratification system proposed by the Korean Society of Thyroid Radiology (KSThR)^2^. The authors included the suspicious LNs in the analysis while excluding the intermediate and probably benign LNs. The CT criteria for identifying suspicious LNs necessitate the presence of at least one of the following characteristics: cystic change, strong enhancement, heterogeneous enhancement, or calcification, all of which are indicative of malignancy radiologically. Notably, two senior radiologists performed well in further distinguishing these suspicious LNs as benign or metastatic, with area under the curve (AUC) values of 0.832 and 0.770, respectively, without significant differences from the ensemble model. However, to the best of our knowledge, there are no reported CT criteria for further classifying LNs that have already been stratified as suspicious. It would improve the understanding of conventional CT features if the authors could provide an elucidation of radiologic criteria employed by the radiologists to carry out this further differentiation of suspicious LNs in PTC.

Moreover, there might be potential selection bias in the studied LNs due to the use of ultrasound (US)-guided fine-needle aspiration (FNA) and washout thyroglobulin examination as a reference for malignancy, even if this method can achieve very high accuracy compared with surgical pathology^3^. As only LNs that could be relocated to US and accessible by US-guided biopsy were enrolled, which is an operator-dependent process compared with CT, we believe that only a proportion of suspected LNs discovered on CT were incorporated in this study. Consequently, this raises the question of whether this ensemble model could be applied to other suspicious LNs that did not undergo FNA but is important to determine the extent of surgery. Notably, it is one of the advantages of CT to evaluate the status of LNs that are not suitable for FNA. From these points of view, we suggest combining FNA and neck dissection pathology as a standard reference for further studies to show whether this model could be applied to all the suspicious LNs detected by CT. Additionally, we were also interested in the percentage of suspicious LNs that underwent FNA and the criteria for selecting LNs for FNA in the prospective settings.

Finally, it was highlighted that the majority of LNs included in the present study had a short-axis diameter 8 mm or less, which is an improvement to a previous deep learning model by Lee *et al*.^4^ in which only LNs with size greater than 8–10 mm were assessed. However, there is another concern about the size of the LNs being examined. Since the information for the short-axis diameter has already been incorporated into radiologic data during segmentation in the deep learning model, LN short-axis diameter reemerged as one of the six clinical risk factors included in the ensemble model. We question the necessity of including short-axis diameter as both radiologic and clinical determinants within the same model.

Overall, an in-depth understanding of the radiological features derived from preoperative contrast-enhanced CT could contribute toward accurately distinguishing between the benign and malignant LNs among the suspicious ones, which can be complementary or even a potential substitute to the US. While we have expressed specific concerns, it is worth clarifying that we do not contest the authors’ conclusions. This useful deep learning model can help in the treatment planning of lateral neck dissection and in avoiding unnecessary FNA in patients with PTC.

## Ethical approval

Not applicable.

## Consent

Not applicable.

## Sources of funding

This work was supported by National Natural Science Foundation of China (82001814 and 82371962); National High Level Hospital Clinical Research Funding (grant number 2022-PUMCH-B-67); 2021 SKY Beijing Imaging Research Fund of the Chinese International Medical Foundation (Z-2014-07-2101); and College Students Innovation Training Program of Peking Union Medical College (2022zglc06011 and 2023zglc06078).

## Author contribution

J.Q. and R.G.: writing the paper; Y. C.: study design. All authors approved the final version of the manuscript.

## Conflicts of interest disclosure

The authors declare that they have no financial conflict of interest with regard to the content of this commentary.

## Research registration unique identifying number (UIN)

Not applicable.

## Guarantor

Yu Chen.

## Data availability statement

This commentary did not involve our data.

## Provenance and peer review

This paper was not invited.
